# Environmental reservoirs of hypervirulent *Clostridioides difficile*: RT078 strains in wastewater and first detection of RT027 in shellfish in Taiwan

**DOI:** 10.1128/spectrum.01377-25

**Published:** 2025-10-27

**Authors:** Te-Wei Yang, Chin-Shiang Tsai, Ya-Ru Li, Yuan-Pin Hung, Pei-Jane Tsai, Bo-Yang Tsai, Jenn-Wei Chen, Wen-Chien Ko, I-Hsiu Huang

**Affiliations:** 1 Department of Internal Medicine, National Cheng Kung University Hospital, College of Medicine, National Cheng Kung University 34912, Tainan, Taiwan; 2 Department of Medicine, College of Medicine, National Cheng Kung University 34912, Tainan, Taiwan; 3 Department of Microbiology and Immunology, College of Medicine, National Cheng Kung University 34912, Tainan, Taiwan; 4 Department of Internal Medicine, Tainan Hospital, Ministry of Health and Welfare 156935 , Tainan, Taiwan; 5 Department of Medical Laboratory Science and Biotechnology, College of Medicine, National Cheng Kung University 34912, Tainan, Taiwan; 6 Institute of Basic Medical Sciences, College of Medicine, National Cheng Kung University 34912, Tainan, Taiwan; 7 Department of Biochemistry and Microbiology, Oklahoma State University Center for Health Sciences 33264, Tulsa, USA; Taichung Veterans General Hospital, Taichung, Taiwan

**Keywords:** *Clostridioides difficile*, wastewater, shellfish, RT027, RT078, environmental transmission, community-associated infection

## Abstract

*Clostridioides difficile* infection is a global health concern, with increasing community-associated cases. Environmental sources may contribute to transmission. This study assessed hypervirulent *C. difficile* in wastewater and shellfish in southern Taiwan. From April to September 2020, water samples were collected from two domestic wastewater treatment plants (WWTPs), one hospital WWTP, and shellfish from an aquafarm in Tainan City. *C. difficile* isolates underwent molecular identification, toxin gene detection, ribotyping, antimicrobial susceptibility, and multi-locus variable-number tandem repeat analysis (MLVA). Ninety-seven *C. difficile* isolates representing 24 ribotypes were classified into 29 distinct strains—4, 13, and 12 from domestic WWTPs, hospital WWTP, and shellfish, respectively. Among 17 WWTP strains, 64.7% were toxigenic. Hypervirulent RT078 family strains (RT598, RT126, and RT127) with Δ39 base-pair deletion in toxin production control protein C gene were detected in WWTPs. One RT027 strain was discovered in clams—the first environmental report of RT027 in Taiwan. MLVA revealed this strain’s association with the American Type Culture Collection reference strain but distinct from other clinical strains. Five hypervirulent isolates harbored Thr82→Ile mutation in DNA gyrase subunit A, conferring high-level fluoroquinolone resistance (minimum inhibitory concentration >32 mg/L) while remaining susceptible to metronidazole and vancomycin. This first detection of hypervirulent RT078 in wastewater and RT027 in shellfish in Taiwan indicates that environmental reservoirs may contribute to community-associated infections, highlighting the need for expanded surveillance.

**IMPORTANCE:**

This study assessed the presence of *Clostridioides difficile* in wastewater and shellfish from southern Taiwan. Water samples were collected from both household and hospital treatment plants, along with clams from a local aquafarm. We identified several strains of *C. difficile*, including types specifically dangerous and resistant to certain antibiotics, such as fluoroquinolones. Notably, the hypervirulent RT027 strain, associated with severe infections in humans, was detected in shellfish, marking the first report of RT027 in environmental samples in Taiwan. Our findings indicate that food and water sources may contribute to the community transmission of *C. difficile*, highlighting the significance of ongoing environmental surveillance.

## INTRODUCTION

*Clostridioides difficile*, a gram-positive, spore-forming, toxin-producing anaerobic bacillus, causes various clinical manifestations, ranging from asymptomatic carriage to infectious diarrhea, pseudomembranous colitis, toxic megacolon, and septic shock (1). *C. difficile* infection (CDI) affected approximately 500,000 patients annually in the United States, resulting in 30,000 deaths annually (2). A meta-analysis estimated the incidence of hospital-onset CDI at 8.3 cases per 10,000 patient-days (3). Globally, the CDI burden increases between 1990 and 2021, with the age-standardized rate of disability-adjusted life years rising from 1.83 to 3.46 per 100,000 and age-standardized death rate rising from 0.10 to 0.19 per 100,000 (4).

Studies have reported an increasing proportion of community-associated CDI (CA-CDI) cases among all CDI cases in recent decades, ranging from 10% to 40% (5–7). The acquisition of toxigenic *C. difficile* spores from the environment or contaminated food is believed to play a crucial role in CA-CDI development (8–11). Recent studies have focused on the potential community sources of *C. difficile*, including animals, seafood, vegetables, and natural environment (8, 12–15).

Wastewater has been identified as a reservoir for multiple enteric pathogens (16), including *C. difficile* (17). Although wastewater treatment plants (WWTPs) are designed to treat sewage and reduce pathogen loads, the spore-forming ability of *C. difficile* enables its survival through treatment and contamination of the surrounding environment (18). Toxigenic *C. difficile* strains have been isolated from WWTP water samples (17–20). Hypervirulent *C. difficile* strains belonging to the RT078 family have been detected in river water in Taiwan (21). However, research on the presence of *C. difficile* in wastewater and water resources in Taiwan remains limited (22). To assess the epidemiology of toxigenic *C. difficile* strains in southern Taiwan’s water resources, we conducted a laboratory-based surveillance at WWTPs and an aquafarm in Tainan.

## MATERIALS AND METHODS

### Isolation of *C. difficile* from wastewater samples and shellfish

**Fig 1:**
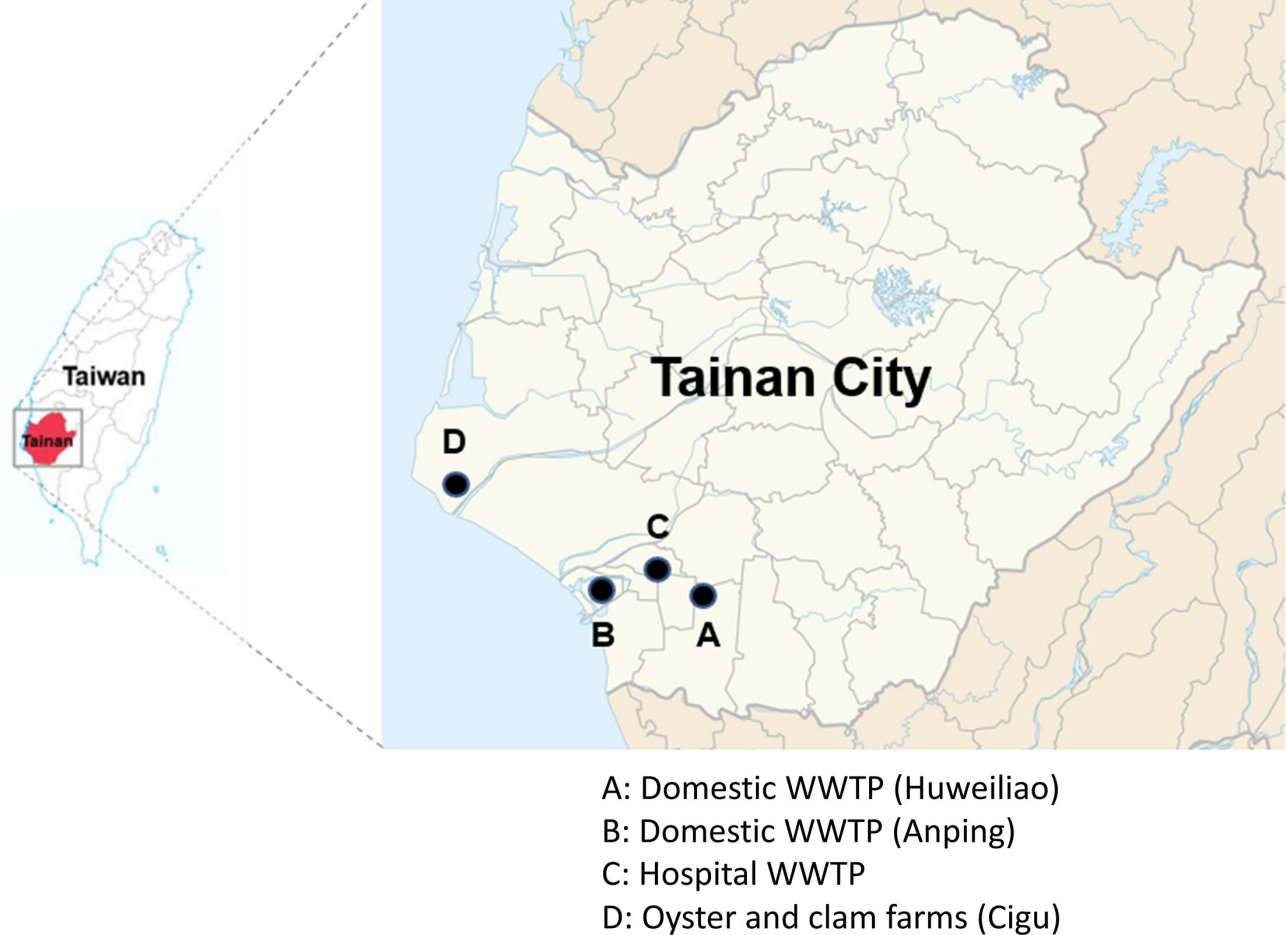
Wastewater and shellfish sampling sites in Tainan City.

This laboratory-based surveillance assessed the presence of hypervirulent *C. difficile* in water samples collected from two domestic WWTPs in Tainan City—Huweiliao (A) and Anping (B)—a 1,300-bed hospital WWTP (C), and an aquafarm (D). WWTP A is located in the most densely populated area of Tainan City, adjacent to an industrial zone. WWTP B is one of the largest WWTPs in southern Taiwan. The sampling sites are illustrated in Fig. 1. Approximately 800 mL of wastewater was collected from both inflow and outflow pipelines, placed in sterile sampling bags (3M; Saint Paul, MN, USA), stored at 4°C, and transported for *C. difficile* isolation.

Wastewater samples were filtered through mixed cellulose ester filter membranes (0.45-µm pore size) to remove granules. Subsequently, the membrane filters were placed in 15-mL tubes containing 8 mL of 1× phosphate-buffered saline (pH 8.0) for suspension and heat shocked at 75°C for 30 min. After centrifugation (3,500 *g*, 10 min, 25°C), the supernatant was discarded, and the pellet was treated with an equal volume of 99% ethanol for 1 h. For *C. difficile* isolation, alcohol-treated samples were centrifuged (3,500 *g*, 10 min, 25°C) to remove residual alcohol. Finally, 200 µL of samples were pre-mixed, streaked onto the BBL CDC Anaerobe 5% sheep blood agar plates (BD, Franklin Lakes, New Jersey), and incubated anaerobically at 37°C for 2 days.

### *C. difficile* colony identification and screening

After anaerobic incubation, plates containing mixed bacterial growth were examined for colonies with *C. difficile* morphology, including irregular edges, ground-glass appearance, and characteristic odor. Suspected colonies were subcultured on fresh BBL CDC Anaerobe 5% sheep blood agar plates to obtain pure isolates. Subsequently, each suspected isolate was confirmed as *C. difficile* using triosephosphate isomerase (*tpi*)-specific polymerase chain reaction (PCR) screening.

### *C. difficile* confirmation through *tpi* gene detection

Genomic DNA was extracted from pure isolates using the MasterPure Complete DNA and RNA Purification Kit (Lucigen, Middleton, WI, USA). Molecular identification was performed using PCR amplification of the *tpi* housekeeping gene, specific for *C. difficile*, using primers Cd *tpi*-F (5′-ATT TAC AGG AGA AGT TTC ACC TCT-3′) and Cd *tpi*-R (5′-GCC CAG ATT GGC TCA TAT GCA AC-3′). The reaction produced a 300 bp product, and only isolates demonstrating positive amplification were confirmed as *C. difficile* and subjected to further characterization.

To obtain *C. difficile* strains, shellfish (clams and oysters) were brushed clean and shucked using sterile knives and hammers. The meat and juice were collected in a sterile filter bag and blended into a homogenate using a sterile blender at full speed for 1 min. Subsequently, the homogenized samples were added to a seafood medium containing proteose peptone (40 g/L), disodium hydrogen phosphate (5 g/L), fructose (6 g/L), sodium chloride (2 g/L), magnesium sulfate (0.1 g/L), and sodium taurocholate (0.1%). The medium, supplemented with cycloserine (500 mg/L) and cefoxitin (15.5 mg/L), was incubated anaerobically at 37°C for 7 days. Following incubation, suspected colonies were selected and confirmed using the same *tpi* gene screening approach. Multiple samples were collected to enhance the production rate. All samples were incubated anaerobically on brain heart infusion (BHI) agar plates or broth supplemented with 5 mg/mL yeast extract and 0.05% L-cysteine (Thermo Fisher Scientific, Waltham, Massachusetts).

### Microbiological analysis of *C. difficile* strains

After incubation, *C. difficile* isolates were subjected to microbiological analyses, including toxin gene detection, PCR ribotyping, antimicrobial susceptibility testing, and sequence of DNA gyrase subunit A (*gyrA*) and *gyrB*. Hypervirulent *C. difficile* isolates were compared with a reference collection of clinical hypervirulent strains. Genomic DNA was extracted using the Presto Mini gDNA Bacteria Kit (Geneaid Ltd, Taiwan) after centrifuging 2 mL of overnight culture at 14,000 *g* for 2 min. The toxin gene profiles of *C. difficile* isolates were assessed using a previously described multiplex PCR approach (23). In summary, our modified method incorporated eight primer sets for the simultaneous detection of *C. difficile* genes: 16S ribosomal DNA (serving as a bacterial internal control), toxin genes (*tcdA* and *tcdB*), binary toxin genes cytolethal distending toxin subunit A (*cdtA*)*/cdtB*, and various toxin production control C (*tcdC*) isoforms. Ribotyping was performed using the QIAxcel capillary electrophoresis system (Qiagen, Hilden, Germany) and QX Alignment Marker 15 bp/3 kb (Qiagen, Hilden, Germany). Ribotypes were confirmed through a two-step analysis, as previously described (23).

### Antimicrobial susceptibility test for *C. difficile* strains

Antimicrobial susceptibility to moxifloxacin, levofloxacin (LVX), ciprofloxacin (CIP), vancomycin (VAN), and metronidazole (MTZ) was examined using E-test strips (Liofilchem, Waltham, Massachusetts). *C. difficile* strains were subcultured 100-fold in fresh BHI broth until reaching an OD600 of 1.0. Subsequently, 200 µL of culture was spread on BHI agar, and E-test strips were placed on each plate and incubated anaerobically overnight. Two *C. difficile* control strains, toxigenic R20291 and non-toxigenic CCUG 37780, were included. Amino acid mutation analyses of *gyrA* and *gyrB* sequences were analyzed using the MultAlin interface.

### Genetic relatedness of hypervirulent toxigenic *C. difficile* strains

The genetic relatedness of hypervirulent *C. difficile* strains, not previously reported in epidemiologic studies of Taiwan’s water resources, was analyzed using multi-locus variable-number tandem repeat analysis (MLVA) (23) and compared with that of previously recognized hypervirulent strains (24, 25). Seven repeat loci (A6cd, B7cd, C6cd, E7cd, F3cd, G8cd, and H9cd) of *C. difficile* were studied. The genetic relatedness between two isolates was determined using the summed tandem repeat difference (STRD), with STRD ≤2 and 10 indicating clonal complex and genetically related cluster, respectively. The results were visualized using a minimum spanning tree.

## RESULTS

Between April and September 2020, 97 *C*. *difficile* isolates representing 24 ribotypes were isolated from three WWTPs and shellfish in Tainan City (Table S1). Based on the collection sites, ribotypes, toxin gene profiles, resistance-associated mutations, and antibiotic susceptibility patterns, these 97 isolates were classified into 29 distinct strains for comparative analysis. Four *C. difficile* strains were isolated from water samples collected from two domestic WWTPs (A and B; Fig. 1) in April 2020 (Table 1). Two of these four strains (50%) were toxigenic and obtained from the inlet pipelines. The HW12F isolate from WWTP A was identified as RT598 of the RT078 lineage and exhibited a toxin gene profile (*tcdA*, *tcdB*, *cdtA*, and *cdtB*) with a Δ39 base-pair (bp) deletion in *tcdC*. The toxigenic strain AP10-1 from WWTP B belonged to RT106 and carried *tcdA* and *tcdB* genes with a wild-type *tcdC*.

**TABLE 1:** Characteristics of four *C. difficile* strains collected from two domestic WWTPs in April 2020^*a*^^,^^*b*^

Isolate code	Sampling location	Pipeline	RT078 lineage	Ribotype	Targeted genes					
					tpi	tcdA	tcdB	cdtA	cdtB	tcdC
HW12F	A	Inlet	+	RT598	+	+	+	+	+	Δ39 bp
AP10-1	B	Inlet	−	RT106	+	+	+	−	−	WT
AP10-2	B	Inlet	−	RT596	+	−	−	−	−	−
AP4-2	B	Outlet, unchlorinated	−	RT010	+	−	−	−	−	−

^*a*^ bp, base pair; WT, wild type.

^*b*^ ‘+’ and ‘–’ indicate presence or absence, respectively. In ribotype tables, ‘+’ denotes strains belonging to the RT078 lineage, while in gene tables, ‘+’ indicates the presence of the corresponding gene.

Thirteen *C. difficile* strains were obtained from the hospital WWTP (WWTP C; Fig. 1) in July 2020 (Table 2). Of these, nine (69.2%) were toxigenic, with five isolated from the inlet pipeline (including one strain with truncated *tcdA*, *tcdB*, and binary toxins), three from the unchlorinated outlet pipeline, and one from the chlorinated outlet pipeline. Notably, three toxigenic strains belonged to the RT078 family—two RT127 strains (15-1 and 15-3) and one RT126 strain (10-1)—all carrying the Δ39 bp deletion in *tcdC*. Additionally, one RT235 strain (32-1) exhibited a Δ18 bp deletion in *tcdC*.

**TABLE 2:** Characteristics of 13 *C. difficile* strains collected from the hospital WWTP in July 2020^*a*^^,^^*b*^

Isolate code	Pipeline	RT078 lineage	Ribotype	Targeted genes					
				tpi	tcdA	tcdB	cdtA	cdtB	tcdC
1-1	Outlet, chlorinated	−	RT713	+	−	−	−	−	−
4-1	Outlet, chlorinated	−	RT002/2	+	+	+	−	−	WT
9-1	Outlet, unchlorinated	−	RT043	+	+	+	−	−	WT
9-2	Outlet, unchlorinated	−	RT647	+	−	−	−	−	−
10-1	Outlet, unchlorinated	+	RT126	+	+	+	+	+	Δ39 bp
13-1	Outlet, unchlorinated	−	RT633	+	+	+	−	−	WT
15-1	Inlet	+	RT127	+	+	+	+	+	Δ39 bp
15-3	Inlet	+	RT127	+	+	+	+	+	Δ39 bp
17-2	Inlet	−	RT462	+	−	−	−	−	−
26-1	Inlet	−	RT713	+	−	−	−	−	−
32-1	Inlet	−	RT235	+	+	+	−	−	Δ18 bp
34-1	Inlet	−	RT AI-83	+	−	+	+	+	WT
37-1	Inlet	−	RT002/2	+	+	+	−	−	WT

^*a*^ WT, wild type; bp, base pair.

^*b*^ ‘+’ and ‘–’ indicate presence or absence, respectively. In ribotype tables, ‘+’ denotes strains belonging to the RT078 lineage, while in gene tables, ‘+’ indicates the presence of the corresponding gene.

Twelve strains were isolated from shellfish collected from an aquafarm in Cigu (site D, Fig. 1) in September 2020 (Table 3). Eight strains (66.7%) were non-toxigenic, whereas only four strains were toxigenic—three from oyster shells and one from clam shells. Among the toxigenic strains, one RT027 strain (CM1A) was identified in clams. Further analysis revealed that 11 of the 12 strains with resistance-associated amino acid mutations in the quinolone resistance-determining regions (Table 4) harbored Ser366→Ala, Ser416→Ala, or both in GyrB. Notably, five strains with Thr82→Ile in GyrA demonstrated high-level resistance to LVX and CIP (minimum inhibitory concentration >32 mg/L) and belonged to RT078 (RT598, RT126, and RT127) and RT027 (Table 3). In this study, all *C. difficile* strains remained susceptible to MTZ (MIC 0.032–1 mg/L) and VAN (MIC 0.125–0.5 mg/L).

**TABLE 3:** Characteristics of 12 *C. difficile* strains collected from the shellfish of an aquafarm in Cigu in September 2020^*a*^^,^^*b*^

Isolate code	Shellfish	RT078 lineage	Ribotype	Targeted genes					
				tpi	tcdA	tcdB	cdtA	cdtB	tcdC
S2-1A	Oyster	−	RT AI-74	+	−	−	−	−	−
S2-2A	Oyster	−	RT010	+	−	−	−	−	−
S3-1A	Oyster	−	RT020	+	+	+	−	−	WT
S5A	Oyster	−	RT638	+	−	−	−	−	−
S5B	Oyster	−	RT590	+	−	−	−	−	−
S8A	Oyster	−	RT607	+	−	−	−	−	−
S9-1A	Oyster	−	RT592	+	−	−	−	−	−
S10-1A	Oyster	−	RT106	+	+	+	−	−	WT
S12A	Oyster	−	RT012	+	+	+	−	−	−
S13-1A	Oyster	−	RT AI-60	+	−	−	−	−	−
S15A	Oyster	−	RT060	+	−	−	−	−	−
CM1A	Clam	−	RT027	+	+	+	+	+	Δ18 bp

^*a*^ WT, wild type; bp, base pair.

^*b*^ ‘+’ and ‘–’ indicate presence or absence, respectively. In ribotype tables, ‘+’ denotes strains belonging to the RT078 lineage, while in gene tables, ‘+’ indicates the presence of the corresponding gene.

**TABLE 4:** Characteristics of 12 *C. difficile* strains with amino acid mutations in the quinolone resistance-determining region (QRDR)^*a*^

Isolate code	Date	Sampling location	Pipeline	Ribotype	QRDR mutation		Antibiotic susceptibility, MIC, mg/L				
					gyrA	gyrB	MXF	LVX	CIP	MTZ	VAN
HW12F	April 2020	A	Inlet	RT598	Thr82→Ile	Ser416→Ala	6	>32	>32	0.032	0.5
9-2	July 2020	C	Outlet, unchlorinated	RT647	–^*b*^	Ser366→Ala	0.38	1	12	0.064	0.38
10-1	July 2020	C	Outlet, unchlorinated	RT126	Thr82→Ile	Ser416→Ala	8	>32	>32	0.047	0.25
15-1	July 2020	C	Inlet	RT127	Thr82→Ile	Ser416→Ala	6	>32	>32	0.125	0.38
15-3	July 2020	C	Inlet	RT127	Thr82→Ile	Ser416→Ala	>32	>32	>32	0.19	0.38
17-2	July 2020	C	Inlet	RT462	–	Ser366→Ala	1	2	4	0.38	0.125
S2-1A	July 2020	D	N/A	AI-74	–	Ser366→Ala Ser416→Ala	0.75	1	6	1	0.38
S5A	July 2020	D	N/A	RT638	–	Ser366→Ala	1	2	4	0.125	0.125
S5B	July 2020	D	N/A	RT590	–	Ser366→Ala	1	2	3	0.19	0.38
S9-1A	July 2020	D	N/A	RT592	–	Ser366→Ala	1	2	2	0.094	0.19
S15A	July 2020	D	N/A	RT060	–	Ser366→Ala	2	4	>32	0.19	0.38
CM1A	September 2020	D	N/A	RT027	Thr82→Ile	–	>32	>32	>32	0.125	0.38

^*a*^ N/A, data not available; MIC, minimum inhibitory concentration; MXF, moxifloxacin; LVX, levofloxacin; MTZ, metronidazole; VAN, vancomycin.

^*b*^ “–” indicate no mutation detected.

**Fig 2:**
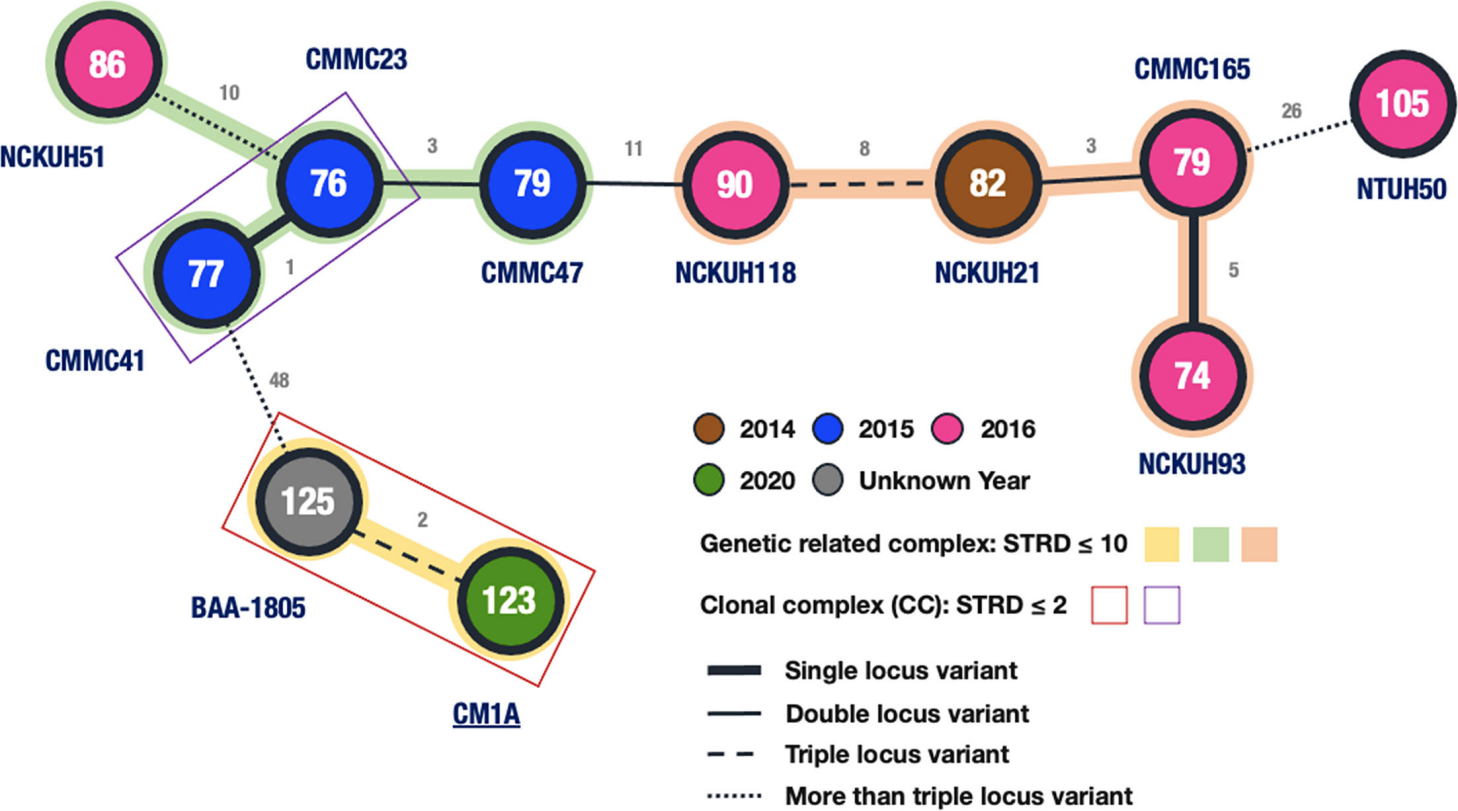
Delineation of microevolution within 11 RT027 strains using MLVA. Minimum spanning tree analyses based on seven microsatellite loci on 11 RT027 strains, including one clam isolate CM1A in this study, one reference strain BAA-1805 purchased from the ATCC, and the rest nine clinical strains from our previous collections (24, 25). The numbers in circles represent the sums of tandem repeats, and the numbers near the lines between circles represent the STRDs.

The RT027 strain (CM1A) in this study represents the first to be isolated from both shellfish and the environment in Taiwan, where its presence has not been previously documented. We compared 11 RT027 strains—one reference strain, nine clinical strains, and CM1A—to determine their genetic relatedness. The MLVA revealed that the clam-derived strain, CM1A, was closely associated with the reference strain American Type Culture Collection (ATCC) BAA-1805 (summed tandem repeat difference [STRD] = 2), placing them within the same clonal complex but distinct from the nine clinical strains collected from three hospitals between 2014 and 2016 (Fig. 2).

## DISCUSSION

This study reports the first detection of hypervirulent RT078 *C. difficile* and RT027 strains from wastewater in Taiwan and environment in Asia, respectively. Both RT027 and RT078 are hypervirulent strains—which may cause higher severity and outcompete endemic strains in the host gut. Studies in the Netherlands have demonstrated that RT078- and RT027-caused CDI has similar severity; however, RT078 strains affect younger populations, are more likely to disseminate in the environment, and are more associated with CA-CDI (26, 27).

CA-CDIs have been increasingly reported in North America and Europe (28), with 18% occurring in patients lacking traditional risk factors, such as prior healthcare exposure, antibiotic use, or acid-suppressing medication (29). These findings support the hypothesis that *C. difficile* can be transmitted environmentally, including zoonotic or foodborne routes, consistent with the “One Health” concept (8, 10, 30, 31). Toxigenic *C. difficile* strains were identified in WWTP-obtained samples in several countries, including Australia, Iran, the United Kingdom, New Zealand, Switzerland, and Canada (18–20, 32–34). A previous environmental surveillance has detected hypervirulent *C. difficile* strains of RT078 family in the rivers of southern Taiwan (21). A multicenter study of clinical *C. difficile* isolates during 2019–2021 demonstrated an increasing trend in the RT078 family (35). Our findings align with these studies and indicate that WWTPs may serve as reservoirs for hypervirulent *C. difficile* strains, facilitating the transmission to animals, seafood, or humans.

The prevalence of *C. difficile* strains varies across wastewater treatment components and geographical regions. In Iran, *C. difficile* was detected in 13.6% of digested sludge samples and 5% of waste stabilization pond samples, but it was absent in both inlet and outlet samples (17). In Western Australia, a survey across 12 WWTPs detected *C. difficile* in 90% and 48% of raw sewage influent and treated effluent samples, respectively, with 55% identified as toxigenic strains (18). Similarly, in southern Switzerland, 24 of 55 *C*. *difficile* (43.6%) strains isolated from 9 WWTPs were toxigenic (34). In our study, 11 of 17 (64.7%) strains from WWTPs were toxigenic. Previous studies identified RT014 (toxin profile A^+^B^+^CDT^-^) as the most common toxigenic strain, whereas RT078 (toxin profile A^+^B^+^CDT^+^) was typically observed (18, 34). In contrast, the most common non-toxigenic strain was RT010. In our study, the strains from WWTPs (toxin profile A^+^B^+^CDT^+^) were identified as members of the RT078 family (RT598, RT126, and RT127).

Hypervirulent *C. difficile* strains are often associated with a high level of antimicrobial resistance, including multidrug resistance (36). Fluoroquinolone resistance in these strains is associated with amino acid mutations in the DNA gyrase subunits (37, 38). In Germany, most drug-resistant isolates (RT001, RT078, RT027, RT014, and RT046) shared the same GyrA mutation (Thr82→Ile), with over 60% of RT027 strains demonstrating fluoroquinolone resistance (39). Widespread fluoroquinolone use created selective pressure, driving convergent resistance in various *C. difficile* ribotypes and giving epidemic strains a competitive advantage in dissemination (39). Our study demonstrated that all five hypervirulent isolates (RT598, RT126, RT127, and RT027) contained an amino acid substitution of GyrA (Thr82→Ile) and exhibited resistance to LVX and CIP. These findings indicate the frequent prescription of fluoroquinolones in clinical settings that may facilitate the selection of hypervirulent *C. difficile* strains.

In our study, one of the six *C. difficile* strains isolated from shellfish in an aquafarm located in southern Taiwan was characterized as RT027. The hypervirulent strain may cause severe CDIs associated with a high mortality rate because of a deletion mutation in *tcdC* and hyperproduction of toxins A and B (40–42). However, RT027-associated CDIs were rarely reported in Asia (43, 44), and its epidemiology and clinical effects in this region remain unclear. A multicenter study in Taiwan (2019–2021) identified 568 *C*. *difficile* isolates, but no RT027 strain was detected (35). To date, in Taiwan, at least three RT027-associated CDI cases have been reported (24, 45, 46), one of which involved a strain susceptible to MFX (MIC 0.5 µg/mL). In contrast, the shellfish-derived RT027 strain in our study was resistant to MFX (MIC >32 µg/mL). MLVAs revealed that the shellfish-derived RT027 strain was clonally associated with the ATCC reference strain but was distinct from clinical RT027 strains (24). The environmental reservoirs of such MFX-resistant RT027 strains warrant further assessment.

This study has certain limitations. First, *C. difficile* isolates were collected from three WWTPs and one aquafarm in Tainan City. Therefore, the findings may not be generalizable to the epidemiology of *C. difficile* in other regions of Taiwan. Additionally, the sample size of *C. difficile* isolates was relatively small, specifically for toxigenic isolates. Second, because the isolates were collected during specific months in 2020, they may not reflect seasonal or long-term variations in *C. difficile* distribution. Third, whole-genome sequencing was not performed. Strain classification was based on ribotyping, toxin gene profiles, resistance-associated mutations, and antibiotic susceptibility testing—which may underestimate the genetic diversity among isolates with the same ribotype. Finally, this study was unable to assess the effects of toxigenic isolates on the community or their association with the occurrence of CA-CDIs.

In conclusion, hypervirulent *C. difficile* strains, including RT078 and RT027, were detected in wastewater and shellfish, respectively, in southern Taiwan. These findings indicate a potential environmental reservoir of toxigenic *C. difficile* that may be associated with CA-CDIs. Comprehensive surveillance studies of *C. difficile* are required to develop effective strategies to prevent CDIs in both the community and healthcare facilities.

## SUPPLEMENTAL MATERIAL

Table S1 spectrum.01377-25-s0001.docx Characteristics of total 97 *Clostridioides difficile* isolates collected from the two domestic wastewater treatment plants.

ASM does not own the copyrights to Supplemental Material that may be linked to, or accessed through, an article. The authors have granted ASM a non-exclusive, world-wide license to publish the Supplemental Material files. Please contact the corresponding author directly for reuse.
