# Supplementary material for: Environmental reservoirs of hypervirulent Clostridioides difficile: RT078 strains in wastewater and first detection of RT027 in shellfish in Taiwan
Source: Microbiol Spectr. 2025 Oct 27;13(12):e01377-25. doi: 10.1128/spectrum.01377-25 (PMC12671137; doi:10.1128/spectrum.01377-25)
Supplement: Table S1 — Characteristics of total 97 Clostridioides difficile isolates collected from the two domestic wastewater treatment plants. [file spectrum.01377-25-s0001.docx]

**Supplementary Table 1. Characteristics of total 97 *Clostridioides difficile* isolates collected from the two domestic wastewater treatment plants—the hospital wastewater treatment plant and shellfish—classified by the ribotyping result, regardless of the location, resistance-associated mutations, and antibiotic susceptibility tests.**

| **Ribotype** |  | ***tpi*** | **RT078-**  **lineage** | ***tcdA*** | ***tcdB*** | ***cdtA*** | ***cdtB*** | ***tcdC*** | **No of isolates** | **Total** |
| --- | --- | --- | --- | --- | --- | --- | --- | --- | --- | --- |
| **RT002/2** |  | **(+)** | **–** | **(+)** | **(+)** | **–** | **–** | **WT** | **3** | **97** |
| **RT010** |  | **(+)** | **–** | **–** | **–** | **–** | **–** | **–** | **2** |  |
| **RT012** |  | **(+)** | **–** | **(+)** | **(+)** | **–** | **–** | **WT** | **1** |  |
| **RT020** |  | **(+)** | **–** | **(+)** | **(+)** | **–** | **–** | **WT** | **1** |  |
| **RT027** |  | **(+)** | **–** | **(+)** | **(+)** | **(+)** | **(+)** | **Δ18 bp** | **2** |  |
| **RT043** |  | **(+)** | **–** | **(+)** | **(+)** | **–** | **–** | **WT** | **1** |  |
| **RT060** |  | **(+)** | **–** | **–** | **–** | **–** | **–** | **WT** | **1** |  |
| **RT106** |  | **(+)** | **–** | **(+)** | **(+)** | **–** | **–** | **WT** | **3** |  |
| **RT126** |  | **(+)** | **(+)** | **(+)** | **(+)** | **(+)** | **(+)** | **Δ39 bp** | **3** |  |
| **RT127** |  | **(+)** | **(+)** | **(+)** | **(+)** | **(+)** | **(+)** | **Δ39 bp** | **55** |  |
| **RT235** |  | **(+)** | **–** | **(+)** | **(+)** | **–** | **–** | **Δ18 bp** | **1** |  |
| **RT462** |  | **(+)** | **–** | **–** | **–** | **–** | **–** | **–** | **6** |  |
| **RT590** |  | **(+)** | **–** | **–** | **–** | **–** | **–** | **–** | **1** |  |
| **RT592** |  | **(+)** | **–** | **–** | **–** | **–** | **–** | **WT** | **1** |  |
| **RT596** |  | **(+)** | **–** | **–** | **–** | **–** | **–** | **–** | **1** |  |
| **RT598** |  | **(+)** | **(+)** | **(+)** | **(+)** | **(+)** | **(+)** | **Δ39 bp** | **1** |  |
| **RT607** |  | **(+)** | **–** | **–** | **–** | **–** | **–** | **–** | **2** |  |
| **RT633** |  | **(+)** | **–** | **(+)** | **(+)** | **–** | **–** | **WT** | **1** |  |
| **RT638** |  | **(+)** | **–** | **–** | **–** | **–** | **–** | **WT** | **1** |  |
| **RT647** |  | **(+)** | **–** | **–** | **–** | **–** | **–** | **–** | **1** |  |
| **RT713** |  | **(+)** | **–** | **–** | **–** | **–** | **–** | **–** | **4** |  |
| **AI-60** |  | **(+)** | **–** | **–** | **–** | **–** | **–** | **–** | **3** |  |
| **AI-74** |  | **(+)** | **–** | **–** | **(+)** | **–** | **–** | **WT** | **1** |  |
| **AI-83** |  | **(+)** | **–** | **–** | **(+)** | **(+)** | **(+)** | **WT** | **1** |  |

WT, wild type; bp, base pair.
